# Progressive Resistance Training and Cancer Testis (PROTRACT) - Efficacy of resistance training on muscle function, morphology and inflammatory profile in testicular cancer patients undergoing chemotherapy: design of a randomized controlled trial

**DOI:** 10.1186/1471-2407-11-326

**Published:** 2011-08-01

**Authors:** Jesper F Christensen, Jesper L Andersen, Lis Adamsen, Birgitte Lindegaard, Abigail L Mackey, Rie H Nielsen, Mikael Rørth, Gedske Daugaard

**Affiliations:** 1 University Hospital Centre for Nursing and Care Research, Copenhagen University Hospital, Blegdamsvej 9, 2100 Copenhagen, Denmark; 2 Department of Oncology, Copenhagen University Hospital, Blegdamsvej 9, 2100 Copenhagen, Denmark; 3 Institute of Sports Medicine, Department of Orthopaedic Surgery M, Bispebjerg Hospital Building 8, Bispebjerg Bakke 23, 2400 Copenhagen, Denmark; 4 Centre for Healthy Ageing, Faculty of Health Sciences, University of Copenhagen, Blegdamsvej 9, 2100 Copenhagen, Denmark; 5 Research Centre of Inflammation and Metabolism, Copenhagen University Hospital, Blegdamsvej 9, 2100 Copenhagen, Denmark

**Keywords:** Testicular cancer, anti neoplasms, resistance exercise, muscle morphology

## Abstract

**Background:**

Standard treatment for patients with disseminated germ cell tumors is combination chemotherapy with bleomycin, etoposide and cisplatin (BEP). This treatment is highly effective, but the majority of patients experience severe adverse effects during treatment and are at risk of developing considerable long-term morbidity, including second malignant neoplasms, cardiovascular disease, and pulmonary toxicity. One neglected side effect is the significant muscular fatigue mentioned by many patients with testicular cancer both during and after treatment. Very limited information exists concerning the patho-physiological effects of antineoplastic agents on skeletal muscle. The primary aim of this study is to investigate the effects of BEP-treatment on the skeletal musculature in testicular cancer patients, and to examine whether the expected treatment-induced muscular deterioration can be attenuated or even reversed by high intensity progressive resistance training (HIPRT).

**Design/Methods:**

The PROTRACT study is a randomized controlled trial in 30 testicular cancer patients undergoing three cycles of BEP chemotherapy. Participants will be randomized to either a 9-week HIPRT program (STR) initiated at the onset of treatment, or to standard care (UNT). 15 healthy matched control subjects (CON) will complete the same HIPRT program. All participants will take part in 3 assessment rounds (baseline, 9 wks, 21 wks) including muscle biopsies, maximum muscle strength tests, whole body DXA scan and blood samples. *Primary outcome*: mean fiber area and fiber type composition measured by histochemical analyses, satellite cells and levels of protein and mRNA expression of intracellular mediators of protein turnover. Secondary outcomes: maximum muscle strength and muscle power measured by maximum voluntary contraction and leg-extensor-power tests, body composition assessed by DXA scan, and systemic inflammation analyzed by circulating inflammatory markers, lipid and glucose metabolism in blood samples. Health related Quality of Life (QoL) will be assessed by validated questionnaires (EORTC QLQ-C30, SF-36).

**Discussion:**

This study investigates the muscular effects of antineoplastic agents in testicular cancer patients, and furthermore evaluates whether HIPRT has a positive influence on side effects related to chemotherapy. A more extensive knowledge of the interaction between cytotoxic-induced physiological impairment and exercise-induced improvement is imperative for the future development of optimal rehabilitation programs for cancer patients.

**Trial Registration:**

Current Controlled Trials ISRCTN32132990.

## Background

Testicular cancer (TC) is the most common form of cancer among young men in western countries [1], and remains the most curable solid tumor with a 10 year survival of 90 to 95% [2]. The standard treatment for patients with advanced germ cell cancer is 3 or 4 cycles of chemotherapy with cisplatin, etoposide and bleomycin (BEP) depending on prognostic factors. The treatment is highly effective, but the majority of patients experience severe adverse effects during treatment, and are at risk of developing considerable long-term morbidity, including second malignant neoplasms, cardiovascular disease, neurotoxicity, nephrotoxicity, pulmonary toxicity, hypogonadism, decreased fertility, and psychosocial problems [3]. In a study by Orre et al. the prevalence of chronic cancer related fatigue was 17.1% among TC patients compared to 9.7% in the general population. Regression analyses showed that poor quality of life (QoL), various psychosocial and somatic problems, and neuroticism were highly associated with presence of chronic cancer related fatigue [4].

Impaired muscular function and significant muscular fatigue are common complaints among TC patients undergoing chemotherapy. The cause of this muscular deconditioning is unknown, and knowledge regarding the effect of antineoplastic drugs on muscle function and morphology is very limited. There is also a lack of knowledge regarding whether TC patients will benefit from exercise training during chemotherapy.

The primary aim of the study is to investigate the effects of BEP-treatment on skeletal muscle, and to examine whether the treatment-induced muscular deterioration can be attenuated or even reversed by high intensity progressive resistance training (HIPRT) initiated on day 1 of the course of antineoplastic treatment. We have chosen a type of resistance exercise program that has proven to induce significant muscular hypertrophy in both healthy and diseased subjects [5]. Furthermore, the long term effect of an exercise rehabilitation program after the course of treatment will be investigated.

## Methods/Design

### Participants and setting

#### Testicular cancer patients

30 patients with disseminated germ cell cancer, scheduled to initiate 3 cycles of BEP-chemotherapeutic treatment at Rigshospitalet, Copenhagen, will be included in the study. Further inclusion and exclusion criteria are listed in table 1. The TC patients will be included and baseline-tested before the start of the first cycle of BEP treatment.

**Table 1:** Eligibility Criteria

Inclusion Criteria
Age: 18-45
Ability to read and understand Danish
Signed informed consent
Exclusion criteria
Presence of any type of current or previous malign disease
Presence of any type of cardiovascular disease (i.e. Cardiomyopathy, Coronary heart disease etc.)
Presence of any type of chronic disease (i.e. Diabetes mellitus, Chronic Obstructive Pulmonary Disease etc.)

#### Healthy control subjects

15 healthy control subjects, matching TC patients in the following parameters: age, BMI and exercise training history, will be included in the study. This intervention-arm will represent the "standard" effect of HIPRT in young men not affected by antineoplastic treatment. The study will be conducted according to the CONSORT (Consolidated Standards of Reporting Trials) statement for non-pharmacological interventions[6], and has been approved by the Regional Ethics committee for the Capital Region (protocol nr: H-1-2010-049) and the Danish Data Protection Agency (jr.nr. 2010-41-5118).

### Recruitment

**Figure 1:**
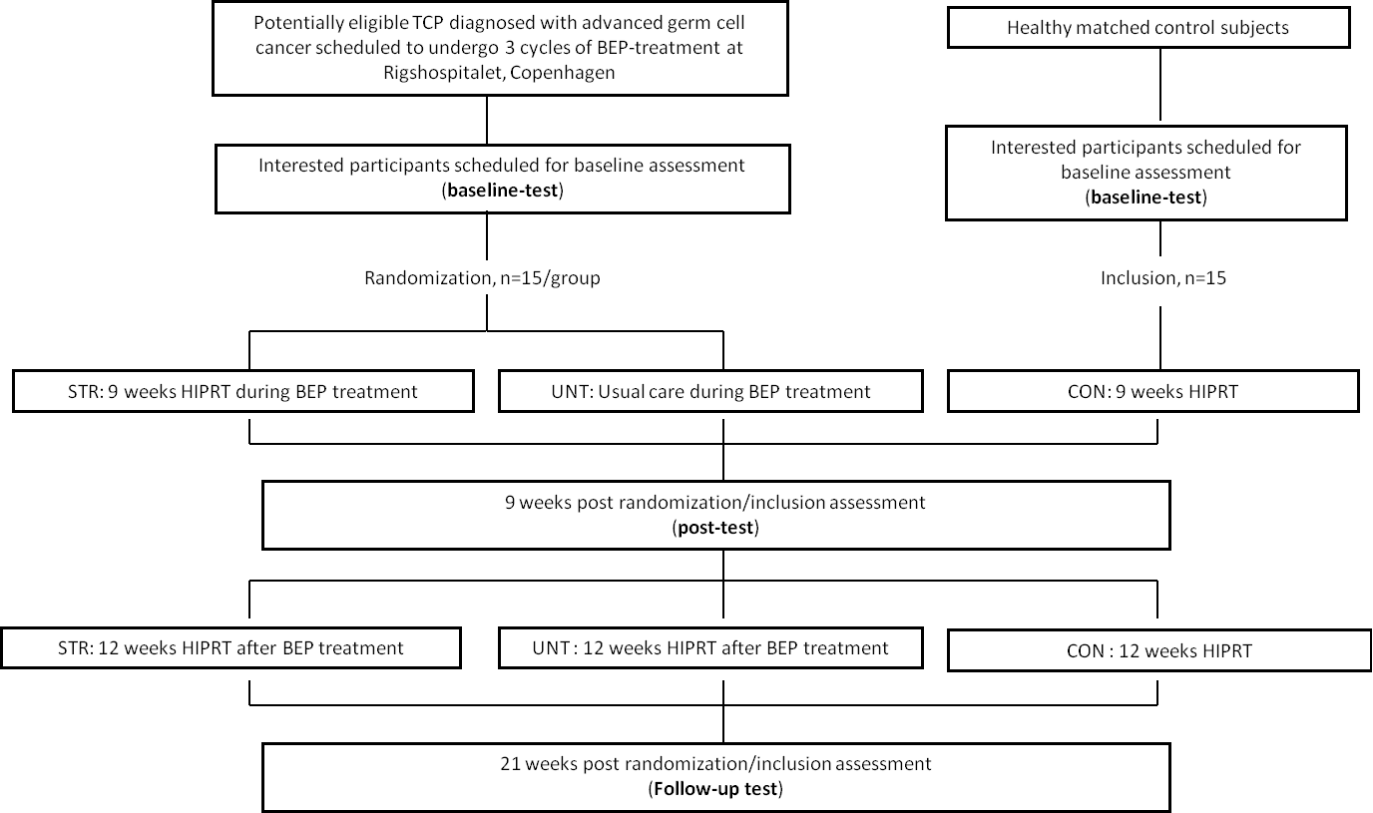
**Study Flow**.

The study flow is presented in Figure 1. TC patients (subjects) will be recruited by clinicians at the Department of Oncology, Rigshospitalet, Copenhagen. The primary attending oncologist will present oral and written information about the study to the eligible participant. Interested participants will be contacted by the study coordinator, who will answer any further questions. The participants will be invited to visit the training facilities, before giving their written consent.

The healthy control subjects will be recruited through announcements in local newspapers and the internet.

### Randomization

Following the baseline test, TC patients will be allocated to either, STRength training (STR, n = 15), receiving a 9-wks HIPRT-program during treatment, or UNTrained (UNT, n = 15), who will receive standard care. The randomization procedure used is a simple adaptive biased-coin randomization. The inclusion criteria (men, aged 18-45, same diagnosis/stage of disease and treatment protocol) secure comparable groups in terms of sex, age and disease profile at baseline, and therefore no stratification will be carried out. The healthy CONtrol subjects (CON, n = 15) will be allocated to HIPRT without randomization.

### Blinding

Blinding of participants in this type of study is not possible since participants will know whether they are exercising or not. Nor are the training instructors blinded, since they will know if they are instructing a patient undergoing chemotherapy or a healthy control subject. However, study personal doing baseline, post-intervention and follow-up assessment will be blinded, as will laboratory personal analyzing invasive biological samples (blood and muscle samples). Only the study coordinator and leading oncologist will know which TC patients are allocated in which groups.

### Treatment

Antineoplastic treatment for TC patients consists of cisplatin 20 mg/m^2 ^and etoposide 100 mg/m^2 ^daily for 5 days and bleomycin 15.000 IU/m^2 ^given weekly. Three cycles of this combination are administered, with every cycle lasting three weeks.

### Intervention

The exercise intervention will be comprised of high intensity progressive resistance training (HIPRT) with 3 training sessions pr week, as previously described by Andersen and Aagaard [5]. In week 1 and 2 the participants will conduct 3 sets of 15 repetitions (reps) at a 15-repetition maximum (RM) intensity to become familiarized with the exercises. From week 3 and onwards, the participants will conduct 4 sets of 10 reps at 10-12 RM intensity. Progression will be assured, such that if the participant can do more than 12 reps, the weight will be increased. All exercises will be performed in a supervised setting to ensure correct technique and proper progression. This type of resistance exercise program has been proven to induce significant muscle hypertrophy in both healthy and diseased subjects [5].

#### Study arm 1: HIPRT for TC patients during and after BEP treatment (STR)

This group will conduct a 9 week HIPRT-program, initiated at day 1 of the treatment, and will be offered a 12 week rehabilitation-program after the course of chemotherapy.

#### Study arm 2: Usual care for TC patients during BEP treatment and HIPRT as rehabilitation following treatment (UNT)

This group will receive standard care for the 9 week period during treatment, and will be offered a 12 week rehabilitation-program with HIPRT after termination of chemotherapy.

#### Study arm 3: HIPRT for healthy matched control subjects (CON)

This group will conduct a 21 week HIPRT program following the same progression as STR.

### Study Endpoints and Assessment

During the course of the study the participants will carry out physical tests, have invasive biological samples taken and complete standardized questionnaires. The schedule for individual data assessment is presented in table 2, and the study endpoints are summarized in table 3.

**Table 2:** Data Assessment Schedule

Data assessment	Baseline test Day - 5-0	Treatment start Day 1	2^nd ^cycle start Day 22	3^rd ^cycle start Day 43	Post interv. test Day 64-66	Follow-up test ~ Day 150
Muscle biopsy	x				x	x
Blood sample	x		x	x	x	x
DXA scan	x				x	x
Strength test	x				x	x
Questionnaires (SF 36 & EORTC)	x		x	x	x	x
Randomization		x				

**Table 3:** Study Endpoints

Muscular morphology
• Mean fiber area
• Fiber type distribution
• Satellite cells
• Intracellular signaling molecules
Body Composition
• Lean Body Mass
• Fat percentage

Muscle strength
• Maximum Voluntary Contraction
• Leg Extensor Power

Systemic effects
• Cholesterol (TCHO, HDL, LDL, Triglycerides)
• Glucose
• Insulin
• Inflammatory markers (CRP, TNF-α, IL-6, IL-18)
• Anti-inflammatory markers (IL-4, IL-10)

Patient reported outcomes
• Health related Quality of Life (SF-36 and EORTC)

The healthy control subjects will carry out the same testing protocol and have the same biological samples taken, but will not receive cancer specific questionnaires.

#### Primary outcome

Muscle biopsies will be collected from m. vastus lateralis using the Bergstrom-technique [7]. The muscle samples are immediately mounted with Tissue-Tek and frozen in isopentane cooled with liquid nitrogen, and stored at -80°C until further investigation. The post intervention biopsy will be obtained at 1 cm either distally or proximally (randomized) to the baseline biopsy.

*Mean fiber area *and *fiber type distribution *will be analyzed as described by Andersen & Aaagaard [5]. Serial sections (10 μm) of the muscle biopsy samples will be cut in a cryostat (-20°C), and routine ATPase histochemistry analysis will be performed after preincubation at pH 4.37, 4.60, and 10.30.

*Number and activation status of satellite cells *in muscle biopsies will be assessed by immunohistochemistry, as previously described by Mackey et al. [8]. The primary antibodies for Pax7, Type I myosin (A4.951), and laminin will be applied to the same section. In this approach, sites of Pax7 antigenicity are visible by light microscopy while fluorescence microscopy on the same section reveals which fibres are type I and type II. The same sections can be used to determine the number of myonuclei associated with type I and type II fibres. Active satellite cells can be distinguished from quiescent with the aid of double staining for satellite cells and Ki67, as described [9].

*Levels of Intracellular Signaling Molecules*: Gene expression of different signalling molecules involved in hypertrophy and atrophy, i.e. Insulin-like Growth Factor 1 (IGF-1) and Atrogin-1, will be measured by real-time Reverse Transcription Polymerase Chain Reaction (RT-PCR), as previously analyzed by our laboratory [10,11].

#### Secondary outcome

Physical function tests will include maximum isometric quadriceps muscle strength test by maximum voluntary contraction (MVC)-measurements using '*Good Strength'-*chair, and maximum muscle power will be assessed by Leg Extensor Power (LEP)-measurements in Nottingham Power-Rig.

*Maximum voluntary contraction*: The participants will be fixated in the Good Strength chair (Metitur Oy, FI-40250 Jyväskylä, Finland. CE certificate: NB ID 0537) with a fixed knee angle of 60 degrees (0 degree = full extension) [12]. Maximum isometric muscle torque will be assessed for both quadriceps (extension) and hamstring (flexion) for both legs. Which leg will be tested first will be randomly selected. A minimum of 3 attempts will be carried out for each leg, and the participants will continue until one attempt is lower than the maximum. The overall maximum attempt (for both legs) will be used as test result.

*Leg Extensor Power*: The participant is seated in the Power Rig (Nottingham Power Rig, Queen's Medical Centre Nottingham, NG7 2UH, United Kingdom) with joint angles as if the participant was rising from a chair. The participants extend one leg as forcefully as possible, and the velocity of the flywheel is measured by an optoswitch and used to calculated the average leg extensor power in the push [13]. Each leg is tested separately, a minimum of 5 attempts is carried out for each leg, however the participant continues until two attempts are lower than the maximum. The overall maximum attempt for both legs is used as the test result.

*Systemic inflammation, lipid and glucose metabolism *will be measured in fasting blood samples. The participants will arrive at the hospital after an overnight fast (minimum 8 hours) and have a 10 ml venous blood sample drawn according to standard clinical guidelines.

Circulating cytokines will be analyzed in plasma using ethylenediaminetetraacetate (EDTA) as an anticoagulant. Plasma is stored at - 80 C until analysis. C-reactive Protein (CRP), Tumor Necrosis Factor alpha (TNF-α), Interleukin-6 (IL-6), Interleukin-18 (IL-18), Interleukin-4 (IL-4) and Interleukin-10 (IL-10) are determined by enzyme-linked immunosorbent assay (ELISA) kits, as previously described by Lindegaard et al [14].

Total cholesterol (TCHO), high density lipoprotein (HDL)-cholesterol, low density lipoprotein (LDL)-cholesterol, triglycerides, glucose and Insulin are measured using standard laboratory procedures.

*Whole Body composition *will be assessed by whole body Dual-Energy X-ray Absorptiometry, (DXA scan) (DPX-IQ Lunar, Lunar Corporation Madison, WI, USA). Transverse scans at 1 cm intervals are made from head to toe measuring the absorption of x-ray beams at two different energy levels as these are sent through the body. Since the different chemical compounds (bone, fat, and fat-free/bone-free mass) in the body absorb the intensity of the X-rays differently, the scan allows for a valid determination of bone mass, fat mass and fat-free/bone-free mass [15].

The DXA scans will be carried out in the morning after an overnight fast. The participants will be asked to standardize their food and water intake on the day prior to scanning to minimize difference in overall hydration as well as extra- and intracellular water distribution as much as possible [16].

*Patient reported outcomes related to Health Related Quality of Life (QoL) *will be assessed be standardized questionnaires: European Organization for Research and Treatment of Cancer Quality of Life Questionnaire (EORTC QLQ-C30) [17] and Medical Outcomes Study Short Form (MOS SF-36) [18]. EORTC QLQ-C30 comprises five functional scales, nine symptom scales or items, and a global health status/quality of life scale. A high score on the functional and global health status/quality of life scale represents a high or healthy level of functioning and high quality of life, while a high score for a symptom scale represents a high level of symptom burden. MOS SF-36 contains eight scales evaluating general health concepts and two summary scales: physical component scale and mental component scale.

### Sample size

Sample size is chosen to ensure significant muscular hypertrophy, evaluated by mean fiber area, in CON, among whom a 15% increase is estimated according to earlier studies.

N > (1,28*√(0.15*0.85))^2^/(0.15^2^) = 9,28. A minimum of 10 is required in each group, but due to clinical experience concerning possible drop out due to medical reasons 15 are included in each group.

### Analytic Plan

1-way repeated measures Analysis Of Variance (RM-ANOVA) will be used to evaluate test-data within groups for assessments at baseline, 9 and 21 weeks. 2-way ANOVA (group × time) will be used to test the effect of HIPRT for TC patients (STR vs. UNT), and BEP-treatment for training response (STR vs. CON) respectively. The equivalent non-parametric tests will be used for mRNA expression and protein levels. Significance level of 0.05 is chosen.

## Discussion

Exercise for cancer patients undergoing treatment with chemotherapy has been shown to improve the patient's physical capacity and to reduce side effects such as fatigue [19]. Recent studies have shown positive effects of exercise on tumor-related outcomes, showing a direct link between exercise induced cellular and systemic changes and reduced tumor progression in breast cancer [20,21]. These results underline findings in large population based studies suggesting that physical activity level is inversely related to mortality in both breast cancer and prostate cancer survivors [22-24]. Consequently, exercise is now being recommended both during and after cancer treatment [25]. However, certain diagnosis groups are clearly overlooked in exercise-oncology research. To our knowledge only 3 exercise studies for cancer patients undergoing chemotherapy have included TC patients - these are data from phase 1, 2 and 3 of the "Body and Cancer" study at our institution [26]. The relative absence of TC patients in exercise-oncology research is probably due to the low incidence rate and good prognosis of this cancer diagnosis. However, a compelling need exists to expand the research related to the acute and late effects of testicular cancer and its treatment, especially with regard to factors that confer an enhanced susceptibility to the toxicities of cisplatin-based chemotherapy. In addition to the extensive degree of treatment induced toxicity resulting in severe acute adverse effects, TC patients face long term side effects, including increased risk of medical disorders such as cardiovascular disease [27-29]. Furthermore, an understanding of the mechanisms that underlie the development of sequelae after cisplatin-based therapy has broader implications because platinum agents are now one of the most widely used groups of cytotoxic drugs worldwide [3].

Knowledge concerning biological effects of cancer treatment on physiological outcomes related to exercise is very limited, and studies elucidating the effect of anticancer treatment on exercise physiological outcomes are in demand [30-35]. It is the main aim of the PROTRACT study to investigate the muscular side effects of BEP treatment in TC patients. Even though it is well established that chemotherapy induces degenerative effects in most body tissues [36,37], only few studies so far have been presented to elucidate the potential damaging effects of antineoplastic agents on the skeletal musculature [38-40]. Effects of Doxorubicin, an antracycline extensively used in breast cancer treatment, have been examined in a number of animal studies, and the myotoxic effect has in part been explained by increased oxidative stress by reactive oxygen species (ROS) causing muscular dysfunction [41,42]. In a recent study Smuder and colleagues showed that exercise protects against this myotoxicity in rats [40,43]. To this point no human studies have assessed intramuscular effects of chemotherapy, however Jones and colleges currently have 2 studies ongoing evaluating muscular effects through collection of muscle biopsies [44,45]. These two studies will analyze muscular morphology in lung cancer- and breast cancer patients respectively, however both studies investigate aerobic exercise interventions, and will mainly focus on the effect on the cardio-respiratory system and the oxygen cascade. Furthermore these studies include only cancer patients who have terminated adjuvant treatment; therefore these patients are not experiencing the concomitant degenerative effects of the anticancer treatment.

We have chosen to investigate the effect of a structured HIPRT program initiated at day 1 of the treatment, to avoid a pre-intervention deterioration. Carrying out an exercise program during the course of a very toxic treatment obviously causes practical challenges due to side-effects like nausea or febrile neutropenia, which may lead to infections. Also TC patients are hospitalized for the first 5 days of each cycle where they receive treatment (chemotherapy and fluids) for 8 to 10 hours per day. This design however, allows us to investigate the direct effects of antineoplastic therapy on skeletal muscle. To minimize confounding factors it is important to eliminate the effect of the tumor itself as much as possible. For this purpose TC patients are optimal, as they have a very responsive tumor and most likely the disease has only minor influence after the first cycle. TC patients, however, may be affected by the presence of hypogonadism, which can significantly affect muscular adaptations to resistance exercise. Up to 50% of leydig cells are lost at unilateral orchiectomy [46], and subnormal levels of testosterone have been found in long term TC survivors [47,48]. In healthy subject it has been shown that suppression of testosterone attenuates the response to resistance training [49], however whether TC patients undergoing chemotherapy experience attenuated exercise response because of treatment induced hypogonadism is currently unknown.

The literature concerning exercise as a concomitant intervention alongside anticancer regimes, i.e. chemotherapy, is constantly growing as an increasing number of studies show positive effects of exercise on physical and psychosocial outcomes. However by far the majority of exercise studies in cancer patients has focused on aerobic training, with limited studies investigating a combined aerobic/resistance program [26] or resistance training alone [50-52]. The relative absence of exercise trials focusing on lean body mass (LBM) is curious, since LBM is regarded an important physiological parameter in overall health profile. When administering chemotherapy in cancer treatment, dose is calculated pr body surface area (in m^2^), only taking into account the patient's height and weight. However, it has been shown that LBM is an independent determinant of 5-Fluorouracil-based toxicity [53], and sarcopenia has been shown to be an independent predictor of toxicity and Time to Progression in breast cancer patients [54]. This indicates the importance of maintaining (or improving) LBM during chemotherapy treatment, hence increasing demands for interventions to improve this specific physiological parameter.

When comparing exercise-oncology studies to exercise studies in healthy subjects, a methodological gap exists in the muscular strength assessment measures. Direct or estimated 1RM measures are almost unanimously used to evaluate maximum muscle strength in exercise-oncology trials. Arguments can be made that the 1RM test mainly evaluates the neurological adaptation to the exercise program, and may overestimate the actual effect on muscle strength due to simple familiarization to the specific exercise. When considering that the main aim of this type of resistance exercise intervention is to increase muscle mass, the 1RM assessment may seem less than optimal. In the PROTRACT study we introduce 2 golden standard measurements for muscle strength and function, MVC and LEP tests respectively, to evaluate the effect on these parameters. These are chosen to ensure valid measurements of muscle strength, both regarding a morphological and a neuromuscular component, whilst minimizing the effect of familiarization.

## Summary

With the increasing interest in the research field of exercise-oncology, more studies are now focusing on the application of exercise as a concomitant intervention alongside anti-cancer therapies. Even though exercise is widely recommended, information concerning the biological/patho-physiological effects of anti-cancer therapies, and especially systemic treatments like chemotherapy, on the skeletal musculature is very limited. To our knowledge no studies have yet reported muscular data from exercise trials in cancer patients undergoing active treatment. Furthermore, there is a distinct need for oncology-exercise trials including male cancer patients, since by far the majority of studies focus on women with breast cancer. To our knowledge the PROTRACT study is the first study designed specifically to address the challenges related to the adverse effects of antineoplastic treatment on physical capacity, muscle mass and muscular function in testicular cancer patients undergoing chemotherapy.

## Abbreviations

BEP: Bleomycin, Etopside, Cisplatin; CON: (healthy) Control group; CRP: C-reactive Protein; DXA: Dual-Energy X-ray Absorptiometry; EDTA: Ethylenediaminetetraacetate; ELISA: Enzyme-linked Immunosorbent Assay; EORTC QLQ-C30: European Organization for Research and Treatment of Cancer Quality of Life Questionnaire; HDL: High Density Lipoprotein; HIPRT: High Intensity Progressive Resistance Training; IGF-1: Insulin-like Growth Factor 1; IL: Interleukin; LDL: Low Density Lipoprotein; LEP: Leg Extensor Power; MOS SF-36: Medical Outcomes Study Short Form-36; MVC: Maximum Voluntary Contraction; QoL: Quality of Life; RM: repetition maximum; ROS: reactive oxygen species; RT-PCR: Reverse Transcription Polymerase Chain Reaction; STR: Strength training group; TC: Testicular Cancer; TCHO: Total Cholesterol; TNF-alpha: Tumor Necrosis Factor-alpha; UNT: Untrained group.

## Competing interests

The authors declare that they have no competing interests.

## Authors' contributions

JFC: conception and design, drafting of manuscript and final approval for publication. JLA: conception and design, drafting of manuscript and final approval for publication. LA: conception and design and final approval for publication. BL: conception and design and final approval for publication. AM: conception and design and final approval for publication. RN: conception and design and final approval for publication. MR: conception and design, drafting of manuscript and final approval for publication. GD: conception and design, drafting of manuscript and final approval for publication.

## Pre-publication history

The pre-publication history for this paper can be accessed here:

http://www.biomedcentral.com/1471-2407/11/326/prepub

## References

[B1] Purdue MP Devesa SS Sigurdson AJ McGlynn KA International patterns and trends in testis cancer incidence Int J Cancer 2005 115 822 827 10.1002/ijc.20931 15704170

[B2] Verdecchia A Francisci S Brenner H Gatta G Micheli A Mangone L Kunkler I Recent cancer survival in Europe: a 2000-02 period analysis of EUROCARE-4 data Lancet Oncol 2007 8 784 796 10.1016/S1470-2045(07)70246-2 17714993

[B3] Travis LB Beard C Allan JM Dahl AA Feldman DR Oldenburg J Daugaard G Kelly JL Dolan ME Hannigan R Constine LS Oeffinger KC Okunieff P Armstrong G Wiljer D Miller RC Gietema JA van Leeuwen FE Williams JP Nichols CR Einhorn LH Fossa SD Testicular cancer survivorship: research strategies and recommendations J Natl Cancer Inst 2010 102 1114 1130 10.1093/jnci/djq216 20585105 PMC2914759

[B4] Orre IJ Fossa SD Murison R Bremnes R Dahl O Klepp O Loge JH Wist E Dahl AA Chronic cancer-related fatigue in long-term survivors of testicular cancer J Psychosom Res 2008 64 363 371 10.1016/j.jpsychores.2008.01.002 18374735

[B5] Andersen JL Aagaard P Myosin heavy chain IIX overshoot in human skeletal muscle Muscle Nerve 2000 23 1095 1104 10.1002/1097-4598(200007)23:7<1095::AID-MUS13>3.0.CO;2-O 10883005

[B6] Boutron I Moher D Altman DG Schulz KF Ravaud P Extending the CONSORT statement to randomized trials of nonpharmacologic treatment: explanation and elaboration Ann Intern Med 2008 148 295 309 18283207 10.7326/0003-4819-148-4-200802190-00008

[B7] Bergström J Muscle electrolytes in man Scand J Clin Lab Invest 1962 68 1 110

[B8] Mackey AL Andersen LL Frandsen U Suetta C Sjogaard G Distribution of myogenic progenitor cells and myonuclei is altered in women with vs. those without chronically painful trapezius muscle J Appl Physiol 2010 109 1920 1929 10.1152/japplphysiol.00789.2010 20930124

[B9] Mackey AL Kjaer M Charifi N Henriksson J Bojsen-Moller J Holm L Kadi F Assessment of satellite cell number and activity status in human skeletal muscle biopsies Muscle Nerve 2009 40 455 465 10.1002/mus.21369 19705426

[B10] Kvorning T Andersen M Brixen K Schjerling P Suetta C Madsen K Suppression of testosterone does not blunt mRNA expression of myoD, myogenin, IGF, myostatin or androgen receptor post strength training in humans J Physiol 2007 578 579 593 17095559 10.1113/jphysiol.2006.122671 PMC2075150

[B11] Jespersen JG Nedergaard A Reitelseder S Mikkelsen UR Dideriksen KJ Agergaard J Kreiner F Pott FC Schjerling P Kjaer M Activated protein synthesis and suppressed protein breakdown signaling in skeletal muscle of critically ill patients PLoS One 2011 6 e18090 10.1371/journal.pone.0018090 21483870 PMC3069050

[B12] Kues JM Rothstein JM Lamb RL Obtaining reliable measurements of knee extensor torque produced during maximal voluntary contractions: an experimental investigation Phys Ther 1992 72 492 501 1409882 10.1093/ptj/72.7.492

[B13] Bassey EJ Short AH A new method for measuring power output in a single leg extension: feasibility, reliability and validity Eur J Appl Physiol Occup Physiol 1990 60 385 390 10.1007/BF00713504 2369911

[B14] Lindegaard B Hansen T Hvid T van HG Plomgaard P Ditlevsen S Gerstoft J Pedersen BK The effect of strength and endurance training on insulin sensitivity and fat distribution in human immunodeficiency virus-infected patients with lipodystrophy J Clin Endocrinol Metab 2008 93 3860 3869 10.1210/jc.2007-2733 18628529

[B15] Lukaski HC Soft tissue composition and bone mineral status: evaluation by dual-energy X-ray absorptiometry J Nutr 1993 123 438 443 8429400

[B16] St-Onge MP Wang Z Horlick M Wang J Heymsfield SB Dual-energy X-ray absorptiometry lean soft tissue hydration: independent contributions of intra- and extracellular water Am J Physiol Endocrinol Metab 2004 287 E842 E847 10.1152/ajpendo.00361.2003 15238354

[B17] Aaronson NK Ahmedzai S Bergman B Bullinger M Cull A Duez NJ Filiberti A Flechtner H Fleishman SB de Haes JC The European Organization for Research and Treatment of Cancer QLQ-C30: a quality-of-life instrument for use in international clinical trials in oncology J Natl Cancer Inst 1993 85 365 376 10.1093/jnci/85.5.365 8433390

[B18] Ware JE Jr Sherbourne CD The MOS 36-item short-form health survey (SF-36). I. Conceptual framework and item selection Med Care 1992 30 473 483 10.1097/00005650-199206000-00002 1593914

[B19] Galvao DA Newton RU Review of exercise intervention studies in cancer patients J Clin Oncol 2005 23 899 909 10.1200/JCO.2005.06.085 15681536

[B20] Murphy EA Davis JM Barrilleaux TL McClellan JL Steiner JL Carmichael MD Pena MM Hebert JR Green JE Benefits of exercise training on breast cancer progression and inflammation in C3(1)SV40Tag mice Cytokine 2011 55 274 279 10.1016/j.cyto.2011.04.007 21600785 PMC3383660

[B21] Hojman P Dethlefsen C Brandt C Hansen J Pedersen L Pedersen BK Exercise-induced muscle-derived cytokines inhibit mammary cancer cell growth Am J Physiol Endocrinol Metab 2011 10.1152/ajpendo.00520.2010 21653222

[B22] Irwin ML McTiernan A Manson JE Thomson CA Sternfeld B Stefanick ML Wactawski-Wende J Craft L Lane D Martin LW Chlebowski R Physical activity and survival in postmenopausal women with breast cancer: results from the women's health initiative Cancer Prev Res (Phila) 2011 4 522 529 10.1158/1940-6207.CAPR-10-0295 PMC3123895 21464032

[B23] Kenfield SA Stampfer MJ Giovannucci E Chan JM Physical activity and survival after prostate cancer diagnosis in the health professionals follow-up study J Clin Oncol 2011 29 726 732 10.1200/JCO.2010.31.5226 21205749 PMC3056656

[B24] Richman EL Kenfield SA Stampfer MJ Paciorek A Carroll PR Chan JM Physical activity after diagnosis and risk of prostate cancer progression: data from the cancer of the prostate strategic urologic research endeavor Cancer Res 2011 71 3889 3895 10.1158/0008-5472.CAN-10-3932 21610110 PMC3107352

[B25] Schmitz KH Courneya KS Matthews C mark-Wahnefried W Galvao DA Pinto BM Irwin ML Wolin KY Segal RJ Lucia A Schneider CM von GV Schwartz AL American College of Sports Medicine roundtable on exercise guidelines for cancer survivors Med Sci Sports Exerc 2010 42 1409 1426 10.1249/MSS.0b013e3181e0c112 20559064

[B26] Adamsen L Quist M Andersen C Moller T Herrstedt J Kronborg D Baadsgaard MT Vistisen K Midtgaard J Christiansen B Stage M Kronborg MT Rorth M Effect of a multimodal high intensity exercise intervention in cancer patients undergoing chemotherapy: randomised controlled trial BMJ 2009 339 b3410 10.1136/bmj.b3410 19826172 PMC2762035

[B27] Huddart RA Norman A Shahidi M Horwich A Coward D Nicholls J Dearnaley DP Cardiovascular disease as a long-term complication of treatment for testicular cancer J Clin Oncol 2003 21 1513 1523 10.1200/JCO.2003.04.173 12697875

[B28] Meinardi MT Gietema JA van der Graaf WT van Veldhuisen DJ Runne MA Sluiter WJ de Vries EG Willemse PB Mulder NH van den Berg MP Koops HS Sleijfer DT Cardiovascular morbidity in long-term survivors of metastatic testicular cancer J Clin Oncol 2000 18 1725 1732 10764433 10.1200/JCO.2000.18.8.1725

[B29] Chaudhary UB Haldas JR Long-term complications of chemotherapy for germ cell tumours Drugs 2003 63 1565 1577 10.2165/00003495-200363150-00004 12887263

[B30] Jones LW Eves ND Haykowsky M Freedland SJ Mackey JR Exercise intolerance in cancer and the role of exercise therapy to reverse dysfunction Lancet Oncol 2009 10 598 605 10.1016/S1470-2045(09)70031-2 19482248

[B31] Jones LW Peppercorn J Exercise research: early promise warrants further investment Lancet Oncol 2010 11 408 410 10.1016/S1470-2045(10)70094-2 20434709

[B32] Ng AV The underrecognized role of impaired muscle function in cancer-related fatigue J Support Oncol 2010 8 177 178 20822036

[B33] Bruera E Cancer-related fatigue: a multidimensional syndrome J Support Oncol 2010 8 175 176 20822035

[B34] Al-Majid S Waters H The biological mechanisms of cancer-related skeletal muscle wasting: the role of progressive resistance exercise Biol Res Nurs 2008 10 7 20 10.1177/1099800408317345 18705151

[B35] Clarkson PM Kaufman SA Should resistance exercise be recommended during breast cancer treatment? Med Hypotheses 2010 10.1016/j.mehy.2010.02.020 20219289

[B36] Chen Y Jungsuwadee P Vore M Butterfield DA St Clair DK Collateral damage in cancer chemotherapy: oxidative stress in nontargeted tissues Mol Interv 2007 7 147 156 10.1124/mi.7.3.6 17609521

[B37] Prado CM Antoun S Sawyer MB Baracos VE Two faces of drug therapy in cancer: drug-related lean tissue loss and its adverse consequences to survival and toxicity Curr Opin Clin Nutr Metab Care 2011 14 250 254 10.1097/MCO.0b013e3283455d45 21415735

[B38] Antoun S Birdsell L Sawyer MB Venner P Escudier B Baracos VE Association of skeletal muscle wasting with treatment with sorafenib in patients with advanced renal cell carcinoma: results from a placebo-controlled study J Clin Oncol 2010 28 1054 1060 10.1200/JCO.2009.24.9730 20085939

[B39] Weber MA Krakowski-Roosen H Schroder L Kinscherf R Krix M Kopp-Schneider A Essig M Bachert P Kauczor HU Hildebrandt W Morphology, metabolism, microcirculation, and strength of skeletal muscles in cancer-related cachexia Acta Oncol 2009 48 116 124 10.1080/02841860802130001 18607877

[B40] Smuder AJ Kavazis AN Min K Powers SK Exercise protects against doxorubicin-induced markers of autophagy signaling in skeletal muscle J Appl Physiol 2011 10.1152/japplphysiol.00429.2011 21778418

[B41] van NK van HA Argiles JM van TS Arts K Gorselink M Laviano A Kegler D Haagsman HP van der Beek EM Direct effects of doxorubicin on skeletal muscle contribute to fatigue Br J Cancer 2009 100 311 314 10.1038/sj.bjc.6604858 19165199 PMC2634729

[B42] Gilliam LA Ferreira LF Bruton JD Moylan JS Westerblad H St Clair DK Reid MB Doxorubicin acts through tumor necrosis factor receptor subtype 1 to cause dysfunction of murine skeletal muscle J Appl Physiol 2009 107 1935 1942 10.1152/japplphysiol.00776.2009 19779154 PMC2793196

[B43] Smuder AJ Kavazis AN Min K Powers SK Exercise protects against doxorubicin-induced oxidative stress and proteolysis in skeletal muscle J Appl Physiol 2011 10.1152/japplphysiol.00677.2010 PMC3075128 21310889

[B44] Jones LW Douglas PS Eves ND Marcom PK Kraus WE Herndon JE Inman BA Allen JD Peppercorn J Rationale and design of the Exercise Intensity Trial (EXCITE): a randomized trial comparing the effects of moderate versus moderate to high-intensity aerobic training in women with operable breast cancer BMC Cancer 2010 10 531 10.1186/1471-2407-10-531 20925920 PMC2965727

[B45] Jones LW Eves ND Kraus WE Potti A Crawford J Blumenthal JA Peterson BL Douglas PS The lung cancer exercise training study: a randomized trial of aerobic training, resistance training, or both in postsurgical lung cancer patients: rationale and design BMC Cancer 2010 10 155 10.1186/1471-2407-10-155 20409311 PMC2888787

[B46] Lackner JE Mark I Schatzl G Marberger M Kratzik C Hypogonadism and androgen deficiency symptoms in testicular cancer survivors Urology 2007 69 754 758 10.1016/j.urology.2007.01.002 17445664

[B47] Brennemann W Stoffel-Wagner B Helmers A Mezger J Jager N Klingmuller D Gonadal function of patients treated with cisplatin based chemotherapy for germ cell cancer J Urol 1997 158 844 850 10.1016/S0022-5347(01)64333-7 9258096

[B48] Gerl A Muhlbayer D Hansmann G Mraz W Hiddemann W The impact of chemotherapy on Leydig cell function in long term survivors of germ cell tumors Cancer 2001 91 1297 1303 10.1002/1097-0142(20010401)91:7<1297::AID-CNCR1132>3.0.CO;2-Z 11283930

[B49] Kvorning T Andersen M Brixen K Madsen K Suppression of endogenous testosterone production attenuates the response to strength training: a randomized, placebo-controlled, and blinded intervention study Am J Physiol Endocrinol Metab 2006 291 E1325 E1332 10.1152/ajpendo.00143.2006 16868226

[B50] Segal RJ Reid RD Courneya KS Sigal RJ Kenny GP Prud'Homme DG Malone SC Wells GA Scott CG Slovinec D'Angelo ME Randomized controlled trial of resistance or aerobic exercise in men receiving radiation therapy for prostate cancer J Clin Oncol 2009 27 344 351 19064985 10.1200/JCO.2007.15.4963

[B51] Courneya KS Segal RJ Mackey JR Gelmon K Reid RD Friedenreich CM Ladha AB Proulx C Vallance JK Lane K Yasui Y McKenzie DC Effects of aerobic and resistance exercise in breast cancer patients receiving adjuvant chemotherapy: a multicenter randomized controlled trial J Clin Oncol 2007 25 4396 4404 10.1200/JCO.2006.08.2024 17785708

[B52] Segal RJ Reid RD Courneya KS Malone SC Parliament MB Scott CG Venner PM Quinney HA Jones LW D'Angelo ME Wells GA Resistance exercise in men receiving androgen deprivation therapy for prostate cancer J Clin Oncol 2003 21 1653 1659 10.1200/JCO.2003.09.534 12721238

[B53] Prado CM Baracos VE McCargar LJ Mourtzakis M Mulder KE Reiman T Butts CA Scarfe AG Sawyer MB Body composition as an independent determinant of 5-fluorouracil-based chemotherapy toxicity Clin Cancer Res 2007 13 3264 3268 10.1158/1078-0432.CCR-06-3067 17545532

[B54] Prado CM Baracos VE McCargar LJ Reiman T Mourtzakis M Tonkin K Mackey JR Koski S Pituskin E Sawyer MB Sarcopenia as a determinant of chemotherapy toxicity and time to tumor progression in metastatic breast cancer patients receiving capecitabine treatment Clin Cancer Res 2009 15 2920 2926 10.1158/1078-0432.CCR-08-2242 19351764

